# The Prevention of Gonorrheal Infection

**Published:** 1909-12

**Authors:** 


A Monthly Review of Medicine and Surgery.

*EDITOR*

WILLIAM WARREN POTTER, M. D.

All communications, whether of a literary or business nature, books for review and xchanges, should be addressed to the editor, 238 DELAWARE AVENUE, BUFFALO, N.Y

VOL. LXV. DECEMBER, 1909. No. 5

### The Prevention of Gonorrheal Infection

CREDE'S method of instillation of a solution of silver nitrate into the eyes of the new born as a prophylactic to ophthalmia is universally recognised by the medical profession after years of use throughout the civilised world, as an almost certain preventive measure even when it can be demonstrated that gonococci are in the vagina at the time of birth. So well is this fact recognised* that it is a routine practice at the present time in all cases of childbirth without reference to the probability of gonorrheal infection. In view of this reliance on prophylactic measures to prevent the all too prevalent destruction of sight, which prevailed before the Credé method was used, it is strange that like preventives are not adopted to prevent infection following clandestine sexual intercourse.

Numerous authorities certify to the fact that if suitable germicides are instilled into the urethra within three hours after coitus (preferably immediately after), the danger of infection is almost naught. Various substances have been used for this purpose including silver nitrate, 2 per cent. protargol, 20 per cent. and albargin, 8 per cent., and while medical journals have made repeated references to social hygiene and societies have been organised to promulgate rules, or issue advice on the dangers of gonorrheal infection, medical men have not informed their patrons nor the public of a safe means of defense. If the same precautions were practised by the physician on the individual following sexual intercourse as are used to prevent gonorrheal ophthalmia, it is reasonably certain that gonorrhea and its fearful results would be reduced to a minimum. Neither prudery, nor a false notion of fostering crime, should play any part in this all important matter. Let the people through the medical profession be made aware of this means of evading this destructive malady.

Prevention is certainly preferable to cure, and the loss of many lives as well as much suffering can thus be prevented. The gonococcus after coitus, if there be any, lodge in the fossa navicularis immediately behind the meatus, and here they can readily be reached with the certainty of destruction if the proper germicidal agent is used and used within three hours after coitus. If a drop of either of the above solutions is allowed,—immediately after coitus preferably,—to flow into the fossa from a syringe or other proper instrument and retained there for 15 or 20 seconds, experience shows they are destroyed. Of course, extreme caution in cleanliness should never be neglected.

The silver salts, protargol or albargin, dissolved in glycerin do not cause the irritation or pain which follows the use of silver nitrate, and can be used by the individual, thus insuring its application within the time limit. The medical profession should become interested in this matter and for humanity's sake, if for no other, make it known to all of their friends, acquaintances and patients. Only by this or some equally feasible and readily applied prophylactic measure can gonorrhea and its havoc be stayed.

Assistant Surgeon E. O. J. Eytinge, U. S. N., in the *Military Surgeon* for August, calls attention to some experiments recently made on the United States War Ships Ranger and Concord, when men allowed shore liberty had been ordered to report at the sick bay immediately, and if they had been exposed to gonorrhea or syphilis to take treatment at once. The instructions posted in the sick bay were as follows: (1) before coming to the sick bay, wash well with water and urinate; (2) in the sick bay wash well with the solution (a 1 to 2,000 solution of mercury bichloride); (3) use the injection and hold it in the canal for three minutes (an injection containing 3 per cent. of protargol and 15 per cent. of glycerin, only about a quarter of a fluid dram being injected, so as to reach not more than the first inch of the urethra; (4) rub the ointment (containing 30 per cent. of calomel) well into the whole penis and leave it on for two hours. Certainly these requirements can not be called onerous; they are simple enough, and the success achieved with them on the Concord and the Ranger seems amply to warrant recourse to them on all the other vessels of the navy.

It is futile to attempt to restrain the men from dangerous intercourse, and it is therefore desirable to try to protect them against the consequences that may happen but for medical intervention. Of 946 men allowed shore liberty at various ports, 256 reported that they had been exposed to venereal infection. These were immediately subjected to prophylactic treatment and not one contracted gonorrhea or syphilis. This severe test would seem to confirm the above observations that if prophylactic measures are instituted within a very few hours after exposure, infection will not occur.

